# Underlying Causes of Death among Adults in the United States, 2013–2017

**DOI:** 10.14218/ERHM.2020.00065

**Published:** 2020

**Authors:** Xin Hu, Yong Lin, Gangjian Qin, Lanjing Zhang

**Affiliations:** 1 Yale School of Public Health, New Haven, Connecticut, USA; 2 Rutgers Cancer Institute of New Jersey, New Brunswick, New Jersey, USA; 3 Department of Biostatistics, School of Public Health, Rutgers University, Piscataway, New Jersey, USA; 4 Department of Biomedical Engineering, University of Alabama at Birmingham, School of Medicine and School of Engineering, Birmingham, Alabama, USA; 5 Department of Pathology, Princeton Medical Center of Princeton, Plainsboro, New Jersey, USA; 6 Department of Biological Sciences, Rutgers University, Newark, New Jersey, USA; 7 Department of Chemical Biology, Ernest Mario School of Pharmacy, Rutgers University, Piscataway, New Jersey, USA

**Keywords:** Causes of death, Trend analysis, Population study, US mortality, Racial disparity

## Abstract

**Background and objectives:**

Overall mortality among U.S. adults has been stable in past years; however, racial disparity influenced 10 leading causes of death or age-specific mortality in Blacks or African Americans. Unfortunately, the trends in sex- and race-adjusted age-standardized cause-specific mortality are poorly understood.

**Methods:**

We here aimed to identify the underlying causes of death (UCD) with sex- and race-adjusted, and age-standardized mortality that has changed in recent years. We extracted the data of UCD from the Multiple Cause of Death database of the Centers for Disease Control and Prevention (CDC). Multivariable log-linear regression models were used to estimate trends in sex- and race-adjusted, and age-standardized mortality of UCD during 2013–2017.

**Results:**

A total of 31,029,133 deaths were identified. Among the list of 113 UCDs compiled by the CDC, there were 29 UCDs exhibiting an upward trend, 33 UCDs exhibiting a downward trend and 56 UCDs with no significant trends. The 2 UCDs with the largest annual percent change were both nutrition related (annual percent change [APC] = 17.73, 95% CI [15.13–20.33] for malnutrition, and APC = 17.49, 95% CI [14.94–20.04] for Nutritional deficiencies), followed by accidental poisoning and exposure to noxious substances. The 4 UCDs with the largest decreasing APC were viral hepatitis (APC = −11.71), chronic and unspecified bronchitis (APC = −8.26), emphysema (APC = −7.11) and human immunodeficiency virus disease (APC = −7.10).

**Conclusions:**

This study thus reports UCDs with changing mortality in recent years after sex- and race-adjustments and age-standardizations. More effort and resources should focus on understanding, preventing and controling the mortality linked to these UCDs. Continuous monitoring of mortality trends is recommended.

## Introduction

Overall mortality among U.S. adults has been stable in past years.^1,2^ Recent works documented the racial disparity in 10 leading causes of death or age-specific mortality in U.S. blacks or African Americans.^3,4^ and disparities of race, age, and sex in the trends of suicide mortality.^5^ However, the trends in sex- and race-adjusted age-standardized cause-specific mortality are largely unknown, despite its significance in public health, policy making and disease prevention. Studies have indirectly shown some rapid-changes in some underlying causes of death (UCD) during 2013–2017,^6,7^ but few studies have focused on the recent trends of other UCD. The recent trends and their changes in our view would also be more useful for current and future policy-making and public health interventions than those during remote years. We therefore aimed to describe the UCD with sex- and race-adjusted, and age-standardized mortality that was changing in recent years (2013–2017).

## Methods

The Multiple Cause of Death database of the Centers for Disease Control and Prevention (CDC) contains mortality and population counts for all U.S. counties.^8^ Its data were extracted from death certificates of all eligible U.S. residents. The age-standardized mortality rates during 2013–2017 were estimated for adults (25+ years) in the U.S. using the U.S. standard population from the year 2000 and the CDC Multiple Cause of Death database (1999–2017).^8^ The age groups of 10-year intervals were used and included the ranges of 25–34, 35–44, 45–54, 55–64, 65–74, 75–84, and 85 years and over. The mortality rate was calculated and shown per 100,000. UCDs were the single, underlying cause of death reported on each death certificate, and here classified using the list of 113 UCDs compiled by the CDC.^1,2^ Age-standardized mortality rates by sex and race (white versus non-white) and multivariable log-linear regression models were used to compute the annual percent change (APC) of sex- and race-adjusted, and age-standardized mortality rates according to the CDC guidelines on using NHCS data.^9^ Briefly, the log-transformed age-standardized mortality rates (as the y) were fit using a simple linear regression model (the x is the year) and adjusted for sex and race. The Stata software (version 15, StataCorp, College Station, TX) was employed for all statistical analyses. This study used de-identified and publicly available data on the deceased subjects. All *P* values were 2-sided and considered significant when <0.05. The trends during 2015–2017 were also computed to assess the potential influence of implementation of the 10th edition of International Classification of Diseases (ICD-10) in 2015.

## Results

**Fig. 1.**
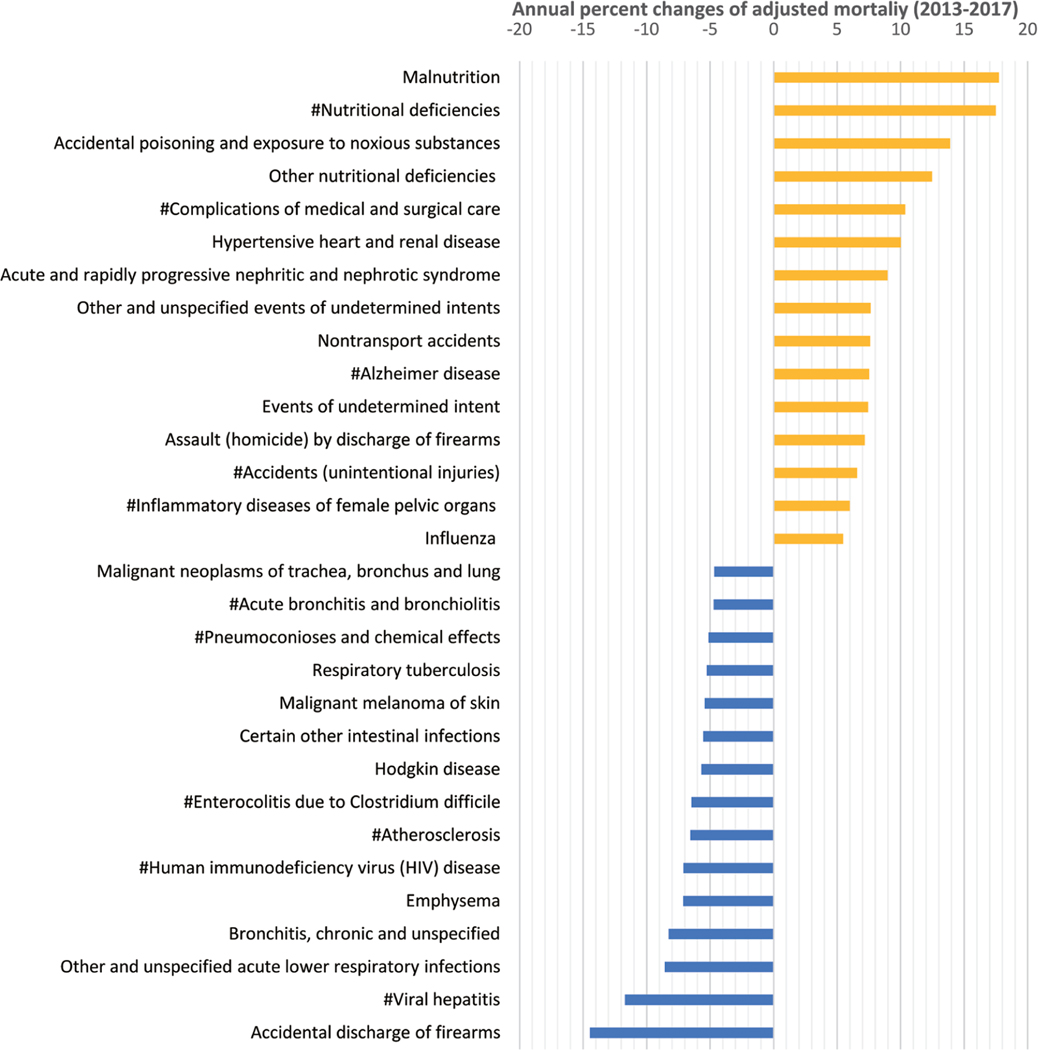
The top 15 underlying causes of deaths for the fastest increasing and decreasing trends in sex- and race-adjusted and age-standardized mortality among adults in the U.S., 2013–2017. The underlying causes of deaths with increasing trends are highlighted in yellow, while those with decreasing trends are highlighted in blue. # indicates a “rankable” cause of death, from the National Center for Health Statistics list of rankable causes of death. The rankable causes are a subset of the 113 selected causes of death.

Per the CDC rules and its data use agreement, any sub-national data representing fewer than ten persons must be statistically suppressed. Among 31,027,401 deaths with un-suppressed mortality and recorded during 2013–2017 (99.9% of the total deaths), there were 14,814,842 women (47.8%) and 26,499,625 whites (85.4%) (Table 1). In the list of 113 UCDs, 29 UCDs had an upward trend in sex- and race-adjusted and age-standardized mortality during 2013–2017 (Table 2 and Fig. 1). These UCDs included, in descending order of APC: Malnutrition/nutritional deficiencies, Accidental poisoning and exposure to noxious substances, Other nutritional deficiencies, Complications of medical and surgical care, Hypertensive heart and renal disease, Acute and rapidly progressive nephritic and nephrotic syndrome, Other and unspecified events of undetermined intent and their sequelae, nontransport accidents, Alzheimer disease, events of undetermined intent, accidents (unintentional injuries), Parkinson disease, alcoholic liver disease and others. Of these 29 UCDs, the 2 UCDs with largest APC were both nutrition-related, followed by accidental poisoning and exposure to noxious substances. A total of 7 accident-related UCDs, 4 heart-related UCDs, 3 hypertension-related UCDs and 2 liver-related UCDs exhibited an increasing sex- and race-adjusted and age-standardized mortality. There were also 33 UCDs exhibiting a downward trend in sex- and race-adjusted and age-standardized mortality (Table 3), and included infection, malignancies and ischemic-heart diseases. There were 56 UCDs with no significant trend, and 15 UCDs exhibiting a statistically suppressed mortality rate. The multivariate sensitivity analysis identified 16 UCDs exhibiting an upward mortality trend and 13 UCDs exhibiting a downward mortality trend during 2015–2017 (data not shown), which all had similar trends during 2013–2017.

## Discussion

Here, we report the UCDs with increasing mortality and those with decreasing mortality among adults in the U.S. during 2013–2017, which were sex- and race-adjusted and age-standardized. Sex- and race-adjustments reduce the biases associated with such disparities in mortality.^4,5,10^ The 2 UCDs with the largest APC were both nutrition related (APC = 17.73 for Malnutrition and APC = 17.49 for nutritional deficiencies), followed by accidental poisoning and exposure to noxious substances. The 4 UCDs with the largest decreasing APC were viral hepatitis (APC = −11.71), chronic and unspecified bronchitis (APC = −8.26), emphysema (APC = −7.11) and human immunodeficiency virus disease (APC = −7.10). Future policy and public health resources should focus on the identified UCDs with growing mortality.

Despite the overall downward trends, cardiovascular diseases, heart diseases, and stroke mortality all exhibited a substantially decelerated downward trend during 2011–2014.^11^ It is therefore possible, but not certain, that some of these UCDs might be linked to increasing mortality in recent years. Moreover, trend analyses on mortality rightfully focused on the leading causes of death.^3,11,12^ Few studies have systematically delineated recent trends of UCDs that exhibit increasing mortality. It is likely effective to decrease overall mortality by identifying and then reversing or neutralizing the increasing morality of certain UCDs. Finally, age-standardized mortality exhibited significantly different trends from those of crude mortality.^10^ Given the variations of mortality trends by race, sex and age, as well as the lack of related data, this study is important for understanding the sex- and race-adjusted age-standardized mortality, and identifying the UCDs linked to changing mortality. This work thus fills the knowledge gap of recent trends in sex- and race-adjusted age-standardized mortality, and provides early data for policy making and specifies public health focus areas for projecting and reducing future mortality among adults in the U.S.

Malnutrition/nutrition deficiency was one of the top-10 leading UCDs among pediatric populations of American Indians/Alaska natives or Asian/Pacific Islanders in 2013^1^ and Hispanic females aged 5–9 years in 2016.^2^ Given its ~17.5% of APC, our data suggest malnutrition/nutrition deficiency will likely become a leading UCD in adults in the near future. The increasing mortality linked to the UCDs of accidents, heart, hypertension and liver are also concerning. Hypertension, for example, was among the top-10 leading UCDs in U.S. adults aged >85 years in 2016, but not in 2013.^1,2^ Future studies should continue monitoring UCD-specific mortality, and better understand and control these upward trends.

Several limitations of this study are noteworthy. First, the UCD on some death certificates may be misclassified or missing altogether. For example, coders more likely include the UCD which the patient recently presented than the ones previously presented. Second, there was a general transition from ICD-9 to ICD-10 coding in 2015. Therefore, some cases may have different ICD-9 and ICD-10 classifications; however, studies have shown that ICD-9 and ICD-10 classifications are overall consistent with each other.^13,14^ In addition, the list of 113 UCDs were unlikely influenced by such a transition because of the relatively broad definition of each UCD (none of them had decimals). Third, we were not able to conduct more-detailed analyses on the subgroups of non-whites owing to the smaller number of cases in select populations such as Asian and Pacific Islanders and blacks. Nevertheless, in some cases, subgroup analyses by race might be important for trend analyses. The reason was that, according to the data use agreement with the CDC, for any unit/cell of the data in a given year, sex and race must be larger than 15. Otherwise, the data cannot be retrieved or used. Finally, there were missing/suppressed data for some UCDs due to the small number of deaths linked to them, and were thus excluded from the analysis.

This study reports the UCDs that are associated with upward or downward trend in sex- and race-adjusted and age-standardized U.S. mortality during 2013–2017. Some of these UCDs have been reported recently and are consistent with our findings,^12^ while others are novel findings. These data will help better prioritize efforts on reducing mortality among U.S. adults. Specifically, our findings shed light on policy making and the allocation of public health resources that should in the future focus on the identified UCDs with growing mortality. Furthermore, these findings may also enable clinical standards to expand interventions and policy-making decisions that are associated with the UCDs of the largest decreasing APC.

### Future directions

Continuous monitoring of mortality trends is warranted. Future works should focus on the causes of rapidly increasing trends in certain UCDs. More importantly, society as a whole should consider approaches to effectively monitor and prevent further increases in deaths associated with these UCDs.

## Figures and Tables

**Table 1.** Baseline characteristics of deaths among adults in the U.S. by year, 2013–2017

	Deaths in 2013 (n = 5,992,801)	Deaths in 2014 (n = 6,049,415)	Deaths in 2015 (n = 6,240,640)	Deaths in 2016 (n = 6,299,895)	Deaths in 2017 (n = 6,446,382)	Total (n = 31,029,133)	*P*
Sex							<0.001
Female	2,891,245	2,900,958	2,990,172	2,988,697	3,044,725	14,815,797	
(%)	48.25	47.95	47.91	47.44	47.23	47.75	
Male	3,101,556	3,148,457	3,250,468	3,311,198	3,401,657	16,213,336	
(%)	51.75	52.05	52.09	52.56	52.77	52.25	
Race							<0.001
Non-White	849,131	867,071	903,395	938,445	970,678	4,528,720	
(%)	14.17	14.33	14.48	14.9	15.06	14.6	
White	5,143,670	5,182,344	5,337,245	5,361,450	5,475,704	26,500,413	
(%)	85.83	85.67	85.52	85.1	84.94	85.4	
Age, year							<0.001
25–34	45,463	47,177	51,517	57,616	60,215	261,988	
(%)	1.79	1.84	1.94	2.15	2.19	1.99	
35–44	69,573	70,996	73,088	77,792	79,796	371,245	
(%)	2.74	2.77	2.76	2.9	2.9	2.82	
45–54	177,724	175,917	174,494	173,516	170,142	871,793	
(%)	7.01	6.86	6.59	6.48	6.19	6.62	
55–64	338,127	348,808	357,785	366,445	372,006	1,783,171	
(%)	13.34	13.6	13.51	13.68	13.53	13.53	
65–74	454,429	471,541	495,016	512,080	531,610	2,464,676	
(%)	17.92	18.38	18.69	19.12	19.33	18.7	
75–84	625,013	624,504	637,566	636,916	657,759	3,181,758	
(%)	24.65	24.35	24.07	23.78	23.92	24.14	
85+	825,198	826,226	859,701	854,462	878,035	4,243,622	
(%)	32.55	32.21	32.45	31.9	31.93	32.2	

P values were calculated using Chi-squared test.

**Table 2.** The underlying causes of death with increasing sex- and race-adjusted and age-standardized mortality among adults in the U.S., 2013–2017

Underlying cause of death^¶^	Mortality in 2013*	Mortality in 2017*	Death during 2013–2017, n	APC (95% CI)	*P*^†^
Malnutrition (E40–E46)	1.37	2.95	25,806	17.73 (15.13 to 20.33)	<0.001
#Nutritional deficiencies (E40–E64)	1.43	3.06	26,788	17.49 (14.94 to 20.04)	<0.001
Accidental poisoning and exposure to noxious substances (X40–X49)	17.28	28.54	230,327	13.89 (10.94 to 16.84)	<0.001
#Complications of medical and surgical care (Y40–Y84,Y88)	1.18	1.75	15,203	10.36 (6.36 to 14.35)	<0.001
Hypertensive heart and renal disease (I13)	1.75	2.86	26,402	10.01 (6.98 to 13.04)	<0.001
Acute and rapidly progressive nephritic and nephrotic syndrome (N00–N01,N04)	0.16	0.23	2,550	8.96 (5.03 to 12.89)	<0.001
Other and unspecified events of undetermined intent and their sequelae (Y10–Y21,Y25–Y34,Y87.2,Y89.9)	1.79	2.25	21,213	7.63 (2.31 to 12.95)	0.011
Nontransport accidents (W00–X59,Y86)	39.14	52.04	501,465	7.59 (5.44 to 9.74)	<0.001
#Alzheimer disease (G30)	36.38	48.01	526,372	7.52 (6.13 to 8.91)	<0.001
Events of undetermined intent (Y10–Y34,Y87.2,Y89.9)	1.91	2.34	22,257	7.44 (2.25 to 12.64)	0.011
#Accidents (unintentional injuries) (V01–X59,Y85–Y86)	53.08	67.47	660,823	6.57 (4.54 to 8.59)	<0.001
#Parkinson disease (G20–G21)	11.26	13.01	140,968	4.67 (3.92 to 5.41)	<0.001
Alcoholic liver disease (K70)	7.88	9.17	102,510	3.97 (2.26 to 5.68)	<0.001
Motor vehicle accidents (V02–V04,V09.0,V09.2,V12–V14,V19.0–V19.2,V19.4)	12.91	14.39	147,771	3.93 (2.59 to 5.26)	<0.001
Transport accidents (V01–V99,Y85)	13.93	15.44	159,358	3.79 (2.56 to 5.02)	<0.001
Heart failure (I50)	27.80	31.56	367,490	3.54 (2.95 to 4.13)	<0.001
Hypertensive heart disease (I11)	15.86	18.78	208,511	2.96 (1.53 to 4.39)	0.001
Intentional self-harm (suicide) by other and unspecified means and their sequelae (*U03,X60–X71,X75–X84,Y87.0)	8.16	9.15	92,679	2.91 (1.35 to 4.47)	0.002
#Intentional self-harm (suicide) (*U03,X60–X84,Y87.0)	16.89	18.27	190,649	2.68 (1.66 to 3.70)	<0.001
Other and unspecified infectious and parasitic diseases and their sequalae (A00,A05,A20–A36,A42–A44,A48–A49,A54–A79,A81–A82,A85.0–A85.1,A85.8,A86–B04,B06–B09,B25–B49,B55–B99)	2.49	2.90	31,630	2.66 (0.70 to 4.63)	0.015
Water, air and space, and other and unspecified transport accidents and their sequelae (V90–V99,Y85)	0.65	0.67	7,376	2.55 (0.39 to 4.70)	0.032
Malignant neoplasms of corpus uteri and uterus, part unspecified (C54–C55)	3.97	4.28	50,862	1.91 (1.42 to 2.39)	<0.001
Other complications of pregnancy, childbirth and the puerperium (O10–O99)	0.49	0.54	4,881	1.87 (0.41 to 3.33)	0.033
#Pregnancy, childbirth and the puerperium (O00–O99)	0.49	0.54	4,976	1.73 (0.24 to 3.23)	0.049
Other diseases of respiratory system (J00–J06,J30–J39,J67,J70–J98)	15.11	16.42	187,819	1.71 (0.75 to 2.67)	0.003
Other and unspecified nontransport accidents and their sequelae (W20–W31,W35–W64,W75–W99,X10–X39,X50–X59,Y86)	6.57	6.85	80,381	1.64 (0.67 to 2.62)	0.004
#Essential hypertension and hypertensive renal disease (I10,I12,I15)	13.17	13.92	161,640	1.41 (0.24 to 2.57)	0.029
#Chronic liver disease and cirrhosis (K70,K73–K74)	15.68	16.80	197,021	1.38 (0.37 to 2.38)	0.015
Other heart diseases (I26–I51)	84.05	87.94	1,052,221	0.91 (0.34 to 1.47)	0.005

Note:

¶ The list of 113 underlying causes of death compiled by the U.S. Centers for Disease Control and Prevention, with the 10^th^ edition of the International classification of Diseases (ICD-10) in parentheses;

* Age-standardized mortality per 100,000 in the U.S.; APC, annual percent change; CI, confidence intervals;

† *P* for sex- and race-adjusted trend in the mortality which was age-standardized using the U.S. standard population of year 2000;

# indicates a “rankable” cause of death, from the National Center for Health Statistics (NCHS) list of rankable causes of death. The rankable causes are a subset of the 113 selected causes of death.

**Table 3.** The underlying causes of death with decreasing sex- and race-adjusted and age-standardized mortality among adults in the U.S., 2013–2017

Underlying cause of death^¶^	Mortality in 2013*	Mortality in 2017*	Death during 2013–2017, n	APC	(95% CI)	*P*^†^
#Viral hepatitis (B15–B19)	3.31	2.13	35,696	−11.71	(−13.31 to −10.11)	<0.001
Bronchitis, chronic and unspecified (J40–J42)	0.26	0.20	2,672	−8.26	(−13.57 to −2.95)	0.007
Emphysema (J43)	3.59	2.78	37,036	−7.11	(−12.14 to −2.07)	0.012
#Human immunodeficiency virus (HIV) disease (B20–B24)	3.17	2.43	31,524	−7.10	(−13.48 to −0.72)	0.042
#Atherosclerosis (I70)	2.83	2.17	30,369	−6.55	(−7.75 to −5.34)	<0.001
#Enterocolitis due to Clostridium difficile (A04.7)	3.32	2.41	35,022	−6.47	(−8.6 to −4.33)	<0.001
Hodgkin disease (C81)	0.48	0.39	5,133	−5.69	(−9.28 to −2.09)	0.006
Certain other intestinal infections (A04,A07–A09)	4.49	3.41	47,250	−5.55	(−7.38 to −3.72)	<0.001
Respiratory tuberculosis (A16)	0.16	0.14	1,808	−5.27	(−10.13 to −0.4)	0.047
Malignant neoplasms of trachea, bronchus and lung (C33–C34)	67.06	56.63	760,393	−4.68	(−6.78 to −2.57)	<0.001
Symptoms, signs and abnormal clinical and laboratory findings, not elsewhere classified (R00–R99)	14.68	11.74	149,792	−4.13	(−6.39 to −1.88)	0.002
Pneumonia (J12–J18)	22.72	19.31	251,383	−4.07	(−5.39 to −2.75)	<0.001
Acute myocardial infarction (I21–I22)	50.11	43.36	566,631	−3.94	(−5.29 to −2.58)	<0.001
#Influenza and pneumonia (J09–J18)	24.25	21.81	273,633	−3.27	(−4.8 to −1.74)	<0.001
Ischemic heart diseases (I20–I25)	158.67	143.56	1,830,094	−2.83	(−3.93 to −1.73)	<0.001
All other forms of chronic ischemic heart disease (I20,I25.1–I25.9)	81.63	73.80	937,255	−2.82	(−4.27 to −1.37)	0.001
Malignant neoplasm of stomach (C16)	4.91	4.44	56,378	−2.58	(−3.48 to −1.68)	<0.001
Other forms of chronic ischemic heart disease (I20,I25)	106.84	98.48	1,242,985	−2.39	(−3.4 to −1.38)	<0.001
Leukemia (C91–C95)	10.02	9.14	112,661	−2.25	(−3.41 to −1.1)	0.001
Malignant neoplasms of kidney and renal pelvis (C64–C65)	5.94	5.44	69,758	−2.22	(−3.14 to −1.31)	<0.001
Malignant neoplasms of lymphoid, hematopoietic and related tissue (C81–C96)	24.60	22.79	282,027	−2.2	(−3.25 to −1.15)	0.001
Non-Hodgkin lymphoma (C82–C85)	8.77	8.13	100,796	−2.2	(−2.99 to −1.41)	<0.001
#Aortic aneurysm and dissection (I71)	4.31	4.00	49,207	−2.14	(−3.29 to −0.98)	0.002
#Malignant neoplasms (C00–C97)	250.98	234.46	2,955,924	−2.01	(−2.4 to −1.63)	<0.001
Malignant neoplasms of colon, rectum and anus (C18–C21)	22.55	21.19	264,027	−1.77	(−2.44 to −1.11)	<0.001
Malignant neoplasm of ovary (C56)	6.11	5.57	70,677	−1.67	(−2.39 to −0.95)	0.001
Other diseases of circulatory system (I71–I78)	8.22	7.80	96,630	−1.59	(−2.48 to −0.7)	0.002
All other diseases (Residual)	136.30	131.52	1,599,086	−1.30	(−2.07 to −0.54)	0.004
Other chronic liver disease and cirrhosis (K73–K74)	7.80	7.68	94,511	−1.26	(−2.39 to −0.13)	0.042
#Septicemia (A40–A41)	16.39	16.13	197,318	−1.15	(−1.89 to −0.41)	0.007
Atherosclerotic cardiovascular disease, so described (I25.0)	25.26	24.65	305,730	−1.12	(−1.83 to −0.42)	0.006
#Diseases of heart (I00–I09,I11,I13,I20–I51)	261.74	254.45	3,133,923	−1.01	(−1.56 to −0.45)	0.002
Major cardiovascular diseases (I00–I78)	341.71	336.23	4,111,472	−0.60	(−0.97 to −0.23)	0.005

Note:

¶ The list of 113 underlying causes of death compiled by the U.S. Centers for Disease Control and Prevention, with the 10^th^ edition of the International classification of Diseases (ICD-10) in parentheses;

* Age-standardized mortality per 100,000 in the U.S.; APC, annual percent change; CI, confidence intervals;

† *P* for sex- and race-adjusted trend in the mortality which was age-standardized using the U.S. standard population of year 2000;

# indicates a “rankable” cause of death, from the National Center for Health Statistics (NCHS) list of rankable causes of death. The rankable causes are a subset of the 113 selected causes of death.

## Abbreviations:

APC: annual percent change
CDC: Centers for Disease Control and Prevention
ICD-10: the 10th edition of International Classification of Diseases
UCD: underlying causes of death
